# Population assessment and habitat associations of the Visayan Hornbill (*Penelopides panini*) in Northwest Panay, Philippines

**DOI:** 10.1186/s40657-021-00303-3

**Published:** 2021-11-29

**Authors:** Holly Isabelle Mynott, David Charles Lee, Rhea Aranas Santillan, Christian Jürgen Schwarz, Benjamin Tacud, Arcel Dryden Fernandez, Daphne Kerhoas

**Affiliations:** 1 Bristol Zoological Society, Bristol Zoo Gardens, College Road, Clifton, Bristol, BS8 3HA UK; 2 School of Applied Sciences, University of South Wales, Alfred Russel Wallace Building, Upper Glyntaff Campus, Pontypridd, CF37 4BB UK; 3 PhilinCon, L.A. Dioso Memorial Public Library, Iloilo-Antique Road, 5712 Pandan, Philippines; 4 PhilinCon Germany, Ruhr-Universität Bochum, Universitätsstraße 150, ND 1/31, 44801 Bochum, Germany

**Keywords:** Distance sampling, Endangered species, GLMM, Hornbill, Point count, Population size, Protected area

## Abstract

**Background:**

Seven out of ten hornbill species in the Philippines are threatened with extinction. Among these is the endangered Visayan Hornbill (*Penelopides panini*), found on the islands of Panay and Negros. Threatened by habitat loss and hunting, its population size is thought to have declined from 1800 individuals 20 years ago to less than 1000. However, a recent study on Negros estimated 3564 individuals across three core forest blocks. This study aims to quantify the Visayan Hornbill population size in and around the Northwest Panay Peninsula Natural Park (NWPPNP) on Panay, the largest contiguous low-elevation forest landscape remaining across its range, and its broad habitat associations across a gradient of environmental degradation.

**Methods:**

Hornbills were surveyed using 10-min distance sampling point counts (*n* = 367) along transects (average length 1.1 km). Environmental variables were recorded along transects, while habitat was classified into primary forest, secondary forest, plantation, or open habitat. Distance software was used to estimate population densities stratified by habitat, with the overall population estimate taken as a mean of habitat density estimates weighted by habitat area. Using generalized linear mixed models, hornbill occurrence was modelled using combinations of nine environmental variables as main and two-way fixed effects.

**Results:**

Surveys covered 204.4 km^2^ of the 374.8 km^2^ Northwest Panay Peninsula. Hornbills were not recorded in plantations or open habitats. Hornbill density was significantly higher in primary forest (17.8 individuals/km^2^ ± 26.9% CV) than in secondary forest (3.7 individuals/km^2^ ± 33.2% CV; *z* = 15.212, *P* < 0.001). The overall population estimate for the NWPPNP and environs is 2109 individuals, and 2673 individuals for the entire Northwest Panay Peninsula. Hornbill presence was best explained by a model including distance from the Park boundary alongside five interaction effects and transect as a random effect. Distance, and the interaction between distance and medium-sized trees were significant predictors of hornbill presence.

**Conclusions:**

Our study evidences the habitat preference of the Visayan Hornbill, highlights the importance of the NWPPNP for the species’ conservation, and provides strong evidence for re-assessing the global population size.

**Supplementary Information:**

The online version contains supplementary material available at 10.1186/s40657-021-00303-3.

## Background

The Philippines is one of the 18 mega-biodiverse countries of the world, and a collective which harbours two thirds of the earth’s biodiversity, while the Philippines itself ranks fourth in levels of global bird endemism (Convention on Biological Diversity 2020). However, Southeast Asia is experiencing a wildlife crisis (Harrison et al. 2016), primarily due to some of the highest deforestation rates in the world (Hughes 2017), and severe hunting pressures (Gray et al. 2018).

Hornbills, as frugivorous birds, play an important role in seed dispersal in tropical forests (Kinnaird and O’Brien 2007). Targeted for domestic consumption and/or the international trade in their casques (Sreekar et al. 2015), their tendency to congregate at fruiting trees and travel long distances makes them particularly vulnerable to hunting pressure (Harrison et al. 2016). Furthermore, as cavity-nesting species, they are also vulnerable to deforestation, as they often rely on old, larger trees in undisturbed forest to breed (Kinnaird and O’Brien 2007). As a result, seven out of the ten hornbill species in the Philippines are considered globally threatened with extinction, among which four are “Endangered” or “Critically Endangered”, while the populations of all ten species are thought to be decreasing (IUCN 2021).

Endemic to the western Visayas of the Philippines (Collar et al. 1999), the globally endangered Visayan (or Tarictic) Hornbill (*Penelopides panini*) is recorded on the islands of Negros and Panay. Small populations may remain on Masbate and Pan de Azucar (BirdLife International 2020), while it is now considered locally extinct on Ticao (Curio 1994; del Hoyo et al. 2001), Guimaras and Sicogon (BirdLife International 2020). The remaining populations exhibit loss of genetic diversity and are probably genetically isolated due to at least 100 km distance between currently available habitats (Sammler et al. 2012). The Visayan Hornbill inhabits dipterocarp forest up to 1100 m a.s.l., occasionally to 1500 m a.s.l., and with a preference for undisturbed habitat, although it does utilise secondary forests (BirdLife International 2020).

As with other hornbills in the region, the Visayan Hornbill is threatened by deforestation and hunting, resulting in an increasingly fragmented and small population (BirdLife International 2020). While natural forest cover (> 30% canopy cover, vegetation > 5 m height) was estimated at ~ 37% and ~ 27% on Panay and Negros, respectively, in 2000, only 5.4% and 2.1% of this was classified as primary forest (Global Forest Watch 2021). Very small forest fragments remain on the species’ other range islands (BirdLife International 2020). Previously estimated at more than 1800 individuals, including 1200 mature individuals, declines in the last 20 years suggest the global population may now comprise less than 1000 individuals, with no subpopulation containing more than 250 individuals (BirdLife International 2020).

Despite ongoing anthropogenic pressures on hornbills, surveys of the Visayan Hornbill have suggested larger population sizes than were previously estimated. Klop et al. (2000) reported an average density of three hornbill nests/km^2^ at Mt. Balabac, Panay, and extrapolated the total breeding population on Panay to be in the range of 750–1500 pairs, under the assumption of 225–450 km^2^ of suitable habitat remaining. A 6-year study by the Philippine Biodiversity Conservation Foundation estimated a population of 3564 individuals across three forest blocks on Negros (Chavez 2020). This island estimate was calculated using a distance sampling point count method and based on an overall lowland forest density of 14 individuals/km^2^. Both these numbers are considerably greater than the last global population estimate for the species (BirdLife International 2020), which is encouraging for the species’ conservation. However, Klop et al.’s (2000) estimate was made 20 years ago, and the exact estimation methods for Chavez’s (2020) study on Negros are unclear.

This study aims to complement the population studies on Negros (Chavez 2020) and the Central Panay Mountain Range (Klop et al. 2000) by quantifying the Visayan Hornbill’s population size within the Northwest Panay Peninsula Natural Park (NWPPNP) and surrounding peninsula on Panay. At 120 km^2^, NWPPNP is the largest remaining contiguous low-elevation forest landscape remaining across the hornbill’s range (BirdLife International 2021a), and where it is reported to be “common” (Curio and Schwarz 2017). The objectives of this study were to (1) quantify the Visayan Hornbill’s population size and generate habitat-specific density estimates across the Northwest Panay Peninsula, using distance sampling, and (2) quantify the hornbill’s habitat associations across a gradient of environmental degradation, from primary forest to open habitat, using Generalised Linear Mixed Modelling (GLMM).

## Methods

### Study site

**Fig. 1:**
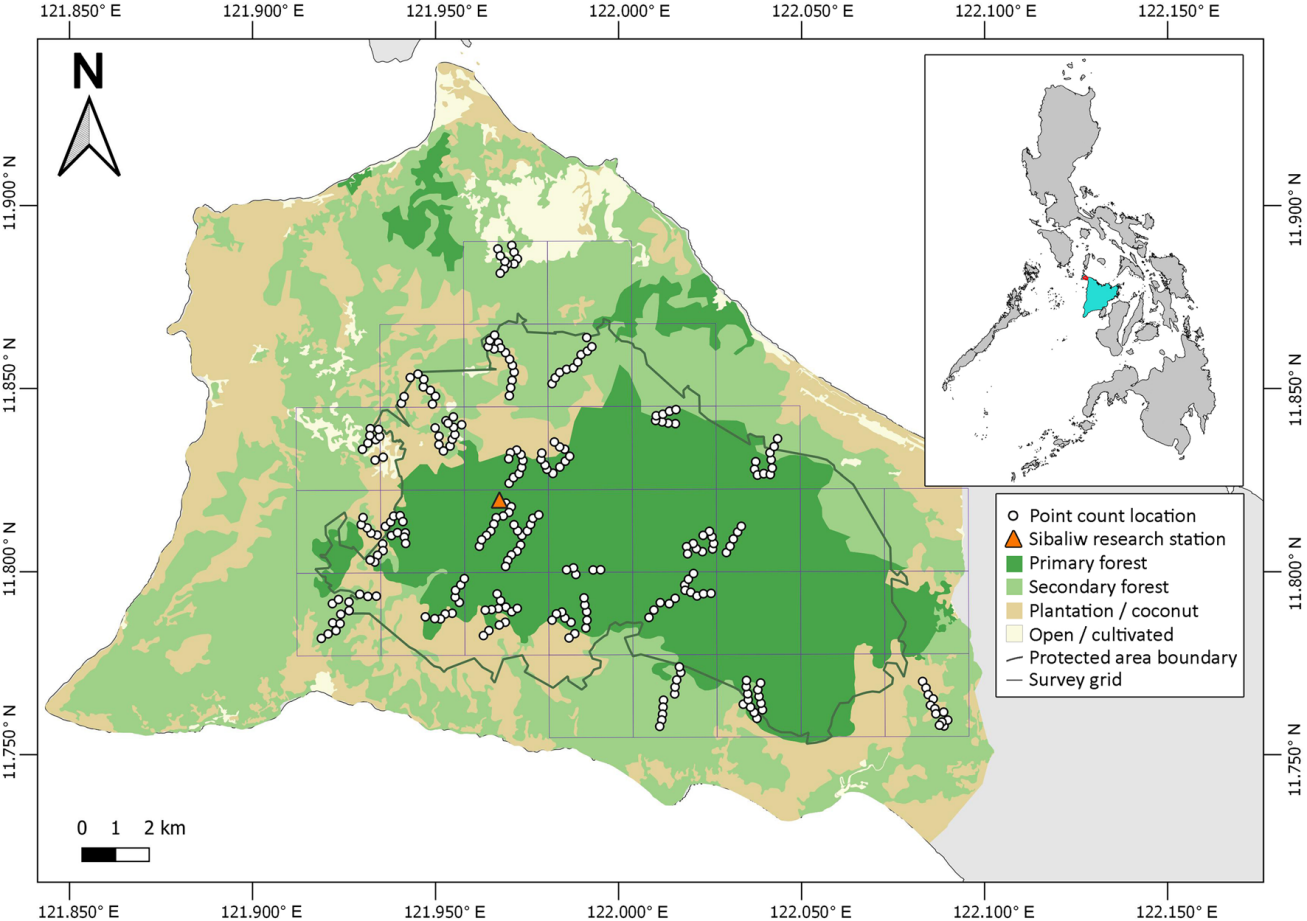
Map of the survey site and its location within the Northwest Panay Peninsula, Panay, Philippines

This survey took place in the Northwest Panay Peninsula Natural Park (NWPPNP; Fig. 1; longitude 122.0003° E, latitude 11.8130° N), a protected area (UNEP-WCMC 2021) since 2002 (PhilinCon 2021). Under the National Integrated Protected Areas System (NIPAS) Act, Philippines, Natural Parks (comparable to IUCN Category II Protected Areas, National Parks) are landscapes not altered significantly by anthropic activity, and managed to maintain their natural, national, or international significance (La Viña et al. 2010). However, while most resource extraction is prohibited, development which is considered “sustainable”, such as renewable energy generation projects, may now be permitted inside buffer zones within the Park’s borders (Congress of the Philippines 2018). The NWPPNP is also an Important Bird and Biodiversity Area (IBA; BirdLife International 2021a), and Key Biodiversity Area (KBA; Key Biodiversity Areas 2020), for which the Visayan Hornbill, as a geographically restricted species, is one of five bird species triggering the site’s KBA classification (Key Biodiversity Areas 2020). The Park (27–875 m elevation) covers 120 km^2^ of tall dipterocarp, limestone karst, lower montane, and bamboo forests, including 25–50 km^2^ of old growth tropical forest (BirdLife International 2021a). It is the largest remaining area of contiguous lowland forest in the Negros and Panay Endemic Bird Area (Key Biodiversity Areas 2020).

While the human population density within the protected area is relatively low (BirdLife International 2021a), there are many settlements located at the forest edge (e.g. La Serna, Paua, Tagosip, Codiong), with some farming legally ongoing inside the Park on plots that existed prior to its establishment (R.A. Santillan pers. obs.), in addition to illegal plots established after the creation of the Park. The Natural Park is managed by the municipal government, the local Department of Environment and Natural Resources (DENR) under NIPAS, and non-governmental organisations (Mogul and Aquino-Ong 2016).

Threats to biodiversity in the NWPPNP include illegal logging and land conversion for slash-and-burn agriculture (kaingin), with some natural forest converted to plantation, and hunting, which is thought to have significantly impacted several bird and mammal species (BirdLife International 2021a). Mining applications encompass the remaining forest cover of the protected area (Key Biodiversity Areas 2020; PhilinCon 2021).

### Survey method

The study site was overlaid with a grid of 2.5 km × 2.5 km cells (*n* = 33), all of which included at least some of the NWPPNP (Fig. 1). Any cell that included shoreline was removed, as the human population is concentrated in coastal areas and most of the habitat there is heavily disturbed. Using a random selection function, 24 cells were identified to survey (72.7% of the study area). However, once on site, three of the selected cells were found to be inaccessible due to very steep terrain. In these instances, the nearest accessible grid cell was surveyed instead. All 24 cells were surveyed once, between 14 January 2020 and 25 March 2020, with surveys repeated in 7 cells (29.2%) during the same period, before access restrictions in response to the coronavirus pandemic halted further repeats. Within each grid cell, 1 to 4 transects of 0.3 to 2.5 km (*x̅* = 1.1 km ± 0.53 SD) were positioned randomly, or along existing narrow trails if the terrain was particularly difficult. Since any trails used were < 1 m width, the positioning of this effort is considered not to have biased results (Cornils et al. 2015). Overall transect effort, not including repeats, was 1 to 3.5 km/cell (*x̅* = 2.3 km ± 0.53 SD).

Distance sampling point counts (Lloyd et al. 2000; Lee and Marsden 2008) were used to survey hornbills along transects. This approach is based on some critical assumptions, which the survey was designed to meet: (1) transect lines are randomly placed with respect to species distribution; (2) birds directly on a point are always detected; (3) birds are detected at their original location before movement; and (4) distances are accurately measured (Buckland et al. 2001). Point counts were situated ≥ 200 m apart along transects, minimising the likelihood of recording the same birds from multiple points (Bibby et al. 1998; Marsden 1999).

A count period of 10 min was used as a compromise between maximizing detection and minimising multiple-counting, and as used previously for hornbill surveys (Marsden 1999). Radial distances to birds were estimated using laser rangefinders (Nikon Aculon AL11 and Volvik V1 models) whenever possible, to the location that the bird was seen at, or from where it was estimated to be calling if it was an auditory-only detection. If a clear line of sight was unobtainable, observers estimated radial distance. Pre-survey training in distance estimation, by sound and sight, ensured there was no between-observer bias in consistently over- or under-estimating distances (Bibby et al. 1998). Point counts were conducted throughout the day, from 08:00 to 18:00. Previous hornbill studies have undertaken surveys throughout morning and afternoon hours (Marsden and Pilgrim 2003; Naniwadekar and Datta 2013), and previous work in the study site has recorded hornbill activity throughout the day (Klop et al. 2000; C.J. Schwarz pers. obs.). Point counts were not carried out in heavy rain or high winds (Marsden and Pilgrim 2003).

### Habitat surveys

The habitat at each point count location was classified into one of four categories: primary forest (old-growth forest), secondary forest, plantation, or open habitat. Forests were classified as primary or secondary based on structural appearance and floristic composition, known history and local knowledge, and supported by satellite imagery (Stouffer et al. 2006). Plantation forest was mostly areas of palm trees, or young, naturally regenerating rainforest interspersed with palms, with the occasional young mahogany plantation. Open habitats included areas of scrub or low-lying ferns with few trees.

Habitat categorisation across the Park was supported by plot-based surveys every 500 m along transects (*n* = 160 plots). Within plots of 20-m diameter (0.03 ha), numbers of tree stems with a diameter at breast height (DBH) of 10–25 cm, 26–99 cm, and ≥ 100 cm were recorded, the latter based on the hornbill’s ecological requirement of large trees for nesting (Klop et al. 2000). The branching architecture of any tree with ≥ 100 cm DBH was described, following Bibby et al. (1998) and as an indication of forest (disturbance) history: Type A, the first major branch is above half of the height of the tree, indicative of growth in primary forest/closed canopy; Type B, the first major branch is below half the height, indicating growth in open canopy; Type C, branching above half the height but with scars where branches have dropped off, indicating growth in regenerating forest; and Type D, vertical branching below half the height, also indicative of growth in regenerating forest. Canopy cover was calculated from four readings (north, east, south, and west facing) from the centre of each plot using a spherical crown densiometer (CSP Forestry concave model). The elevation of each plot and its distance from the Park’s boundary was recorded, the latter as a proxy for accessibility and potential anthropic disturbance.

### Data analysis

To categorise habitat across the remaining peninsula outside the study points, a dataset of land cover was used (PhilGIS 2020), which categorised land into 13 categories, from closed canopy primary forest to built-up land containing human settlements or industry. These areas were re-categorised into the same 4 categories as measured in our survey, based on mapping our measured habitat variables against the PhilGIS dataset, using QGIS version 3 (QGIS Development Team 2021) to compare datasets.

Hornbill survey data were analysed using conventional distance sampling in Distance software version 7.3 (Thomas et al. 2010), modelling the decline in an animal’s detection probability as (radial) distance from the observer (survey point) increases (Buckland et al. 2001). Where group size was uncertain, a habitat-specific mean group size taken from visual detections was used (Lee and Marsden 2008). Detection data were modelled at different right-truncation distances (60 to 105 m) and distance intervals (automatically and manually selected). A series of detection functions (uniform, half-normal, and hazard rate) and expansion terms (cosine, simple and Hermite polynomials) were applied to each model, sequentially; Akaike’s Information Criterion (AIC) was used to select the final candidate model among those with the same truncation distance (Buckland et al. 2001). Distance data were stratified by habitat, with overall population density and size estimated as a mean of habitat density estimates weighted by habitat area. Detection functions were estimated for all data pooled across habitats and for each habitat separately. Model goodness-of-fit across truncation distances was analysed using Kolmogorov–Smirnov and Cramér-von Mises (with cosine weighting function) tests (Buckland et al. 2001) alongside those distances that generated estimates with the lowest coefficients of variation (Kinnaird et al. 1996). These assessments were supplemented by chi-square goodness-of-fit tests, although recognising that these are influenced by the cut-points and distance intervals selected for the model. If the fit of detection functions was similar for pooled data and separate habitats, then the model with the lower AIC was preferred. Quantile–quantile (Qq)-plots were used to help diagnose any departure of the data from a fitted model and refine the modelling process iteratively. Two-sample *z*-tests were used to analyse differences between habitat-specific density estimates (Thomas et al. 2010), and using the formula:

$$z = (mean1 - mean2) / \surd {\{se(mean1)\}}^{2} + {\{se(mean2)\}}^{2}.$$

A generalized linear mixed modelling (GLMM) procedure was used to model hornbill presence-absence against the environmental variables (Table 1). GLMMs were constructed in R software (R Development Core Team 2017) using the package “lme4” (Bates et al. 2015). Models were fitted with a binomial error family and logit link function (Aarts et al. 2021). Scaled explanatory variables were used since the original variables were measured on different scales. The package “MuMIn” (Barton 2009) was then used to determine models of best fit based on AIC corrected for small sample sizes (AICc; Burnham and Anderson 2004). Due to the maximum number of terms permitted in this process (*n* = 31), a series of GLMMs were constructed: one with all possible main fixed effects; and nine, two-way fixed effects models, each in turn with one habitat variable as an interaction effect with the other eight habitat variables. All models included transect as a random effect. Significant effects from these models were then combined in a final GLMM. Since Akaike weights, as a measure of model parsimony, were low for the top ranked models, model average parameters were used (Burnham and Anderson 2004). The package “MuMIn” (Barton 2009) was also used to quantify model fit (*R*^2^), with variances derived from the delta method (Nakagawa et al. 2017), and marginal *R*^2^ and conditional *R*^2^ representing the variance explained by all fixed effects and the full model (fixed and random effects), respectively.

**Table 1:** Descriptives of the explanatory variables used to model hornbill presence-absence

Variable	Abbreviation	Mean ± SE	Range
Distance of survey point from the Park’s boundary (m)	Distance	1165.4 ± 123.22	− 2058–3960^a^
Elevation (m)	Elevation	335.7 ± 14.47	33–907
Canopy cover (%)	Canopy cover	68.2 ± 2.10	0–100
Number of small trees; DBH 10–25 cm	Number of small trees	26.9 ± 1.54	0–104
Number of medium-sized trees; DBH 26–99 cm	Number of medium trees	19.9 ± 1.70	0–220
Number of large trees; DBH ≥ 100 cm	Number of large trees	2.5 ± 0.18	0–9
Proportion of large trees with branching Type A	Proportion branching type A	0.4 ± 0.03	0–1.0
Proportion of large trees with branching Type B	Proportion branching type B	0.2 ± 0.02	0–1.0
Proportion of large trees with branching Types C and D	Proportion branching type C&D	0.2 ± 0.03	0–1.0

^a^ Negative distances relate to points outside the Park’s boundary

## Results

In total, surveys of the NWPPNP and environs covered 204.4 km^2^ (54.5%) of the 374.8 km^2^ Northwest Panay Peninsula, which is delineated to the east by the north–south road from Kalibo and Caticlan to Pandan (easternmost point: longitude 121.1032° E, latitude 11.7503° N). Within the peninsula, 104.7 km^2^ was classified as primary forest, 221.6 km^2^ as secondary forest, 33.1 km^2^ as plantation and 15.3 km^2^ as open area. Based on the data collected through remote spatial resources and habitat plots, primary forest covers about 48% (98 km^2^) of the NWPPNP, secondary forest 49% (99 km^2^) and other areas 3% (7 km^2^), most of which is palm plantation.

### Density and population estimates

In total, 274 points were surveyed, with 93 of the points surveyed twice (*n* = 367). Of these, 111 points were in habitat classified as primary forest, 102 in secondary forest, 39 in plantation, and 22 in open areas (Table 2). Visayan Hornbills were recorded 54 times (mean group size = 1.3 ± 9.1% CV), and never in plantations or open habitats.

**Table 2:** Habitat-specific density and population estimates for Visayan Hornbill in the Northwest Panay Peninsula

Habitat	Habitat area (km^2^)	Total effort^a^	Number of encounters (total number of individuals)	Density estimate ± % CV	Population estimate ± % CV^b^
Primary forest	104.7	165 (111)	31 (43)	17.8 ± 26.9	1861 ± 26.9 (1104–3135)
Secondary forest	221.6	125 (102)	12 (15)	3.7 ± 33.2	813 ± 33.2 (429–1542)
Plantation	33.1	49 (39)	0 (0)	0	0
Open	15.3	28 (22)	0 (0)	0	0

^a^Numbers of points are in parentheses

^b^95% confidence intervals are in parentheses. Estimates are given for the data right truncated at *w* = 95 m

**Fig. 2:**
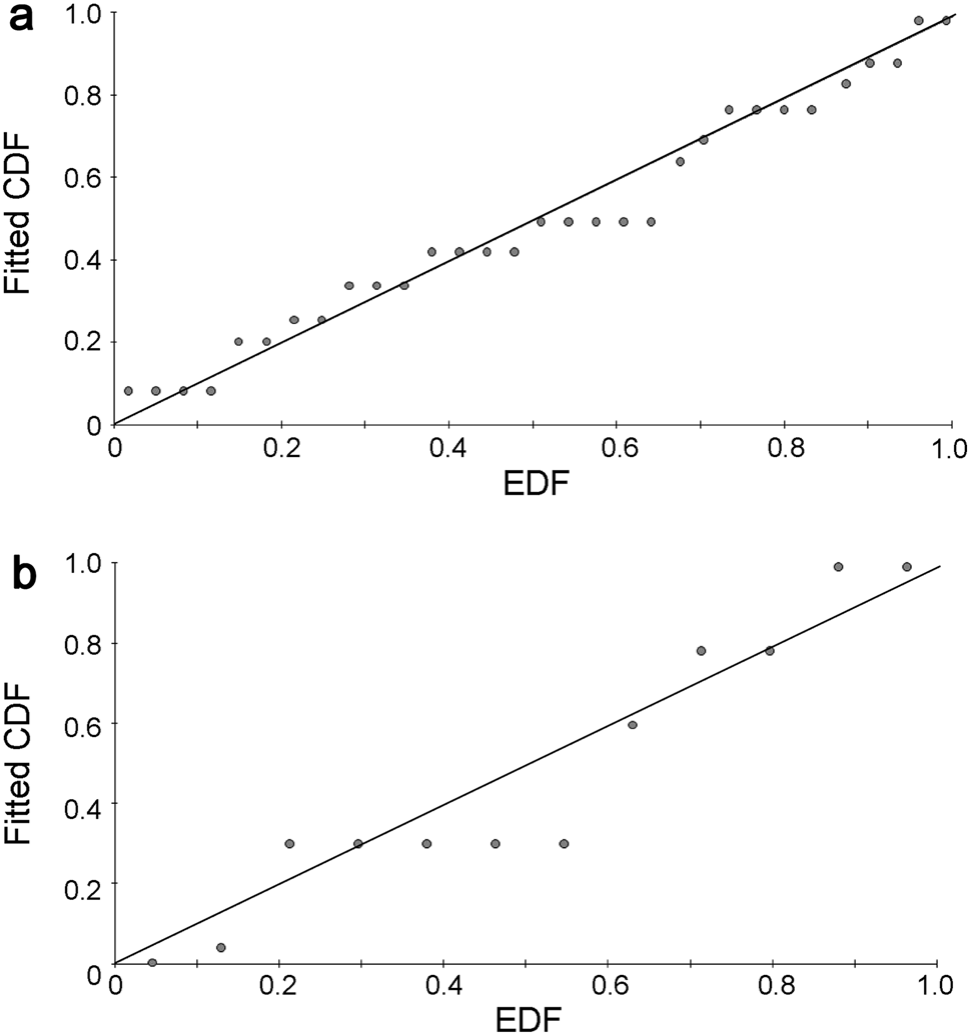
Q-q plots (empirical distribution function, EDF, plotted against fitted cumulative distribution function, CDF) at 95 m right truncation for **a** primary forest and **b** secondary forest

Generally, habitat-based detection functions, rather than an overall detection function, better fitted the distance data (AIC_habitat_ < AIC_overall_; mean ∆AIC =  + 2.6 ± 1.62 SE), indicating habitat-specific detectability. All overall models were best fitted with one parameter and habitat models with two parameters, except 60 m truncation models, which were fitted with two and zero parameters, respectively. Based on goodness-of-fit statistics, and alongside model parsimony, estimation precision and Qq-plots (Fig. 2), a 95 m right truncation with habitat detection functions was considered to best fit the data: primary forest, *D* = 0.181, *P* = 0.260; *C*^2^ = 0.063–0.078, 0.500 < *P* ≤ 0.600; *χ*^2^ = 4.098, *P* = 0.251; secondary forest, *D* = 0.306, *P* = 0.210; *C*^2^ = 0.097–0.123, 0.300 < *P* ≤ 0.400; *χ*^2^ = 5.196, *P* = 0.268). Both primary and secondary forest data were modelled with a uniform detection function; in the case of primary forest, with a cosine adjustment term. There were no detections of hornbills < 20 m from points in primary forest. At 95 m truncation, the probabilities of detection were 0.517 ± 10.7% CV and 1.0 ± 0.0% CV in primary and secondary forests, respectively (Additional file 1: Table S1).

Estimated hornbill density was significantly higher in primary forest (17.8 individuals/km^2^ ± 26.9% CV) than in secondary forest (3.7 individuals/km^2^ ± 33.2% CV; *z* = 15.212, *P* < 0.001). Using these densities generated habitat-specific population estimates for the NWPPNP of 1737 individuals (1031–2927 95% CIs) in primary forest and 371 individuals (196–704 95% CIs) in secondary forest. The overall population estimate, weighted by habitat area and pooled across all habitat strata, for the NWPPNP and its immediate surroundings is 2109 ± 22.9% CV (1350–3294 95% CIs). For the entire Northwest Panay Peninsula (374.8 km^2^), the hornbill population is estimated at 2673 individuals ± 21.3% CV (1767–4045 95% CIs); 1861 individuals (1104–3135 95% CIs) in primary forest and 813 (429–1542 95% CIs) in secondary forest (Table 2).

### Habitat associations

Of the 160 habitat plots, 77 were in primary forest, 49 in secondary forest, 22 in plantations and 12 in open habitat. Visayan Hornbills were recorded in 16 of the 24 grid cells, giving a landscape naive occupancy of 66.7%.

Among the candidate predictor variables, small trees, and the proportion of large trees with branching type A were not included in the top-ranked models, which all comprised one fixed main effect (distance from the Park boundary) and up to six interaction effects (Table 3). Final model fit was moderate ($R_{{\text{m}}}^{2}$ = 34.4%) and improved with the inclusion of transect (variance = 1.179, SD = 1.086, 22 groups) as a random effect ($R_{{\text{c}}}^{2}$ = 44.5%). Model averaging identified two variables as significant predictors of hornbill presence in the Park: distance from the Park boundary (positive), and the interaction between medium-sized trees and distance from the Park’s boundary (negative; Table 4). The positive interactions between distance and elevation, and medium-sized and large trees contributed marginally to explaining hornbill presence.

**Table 3:** Generalized linear mixed models of best fit

Model	ΔAICc	Akaike weight	$R_{{\text{m}}}^{2}$	$R_{{\text{c}}}^{2}$
Distance + Distance:Elevation + Distance:Number of medium trees + Number of large trees:Number of medium trees + Number of large trees:BhB + BhB:BhCD + Transect	0.00	0.07	0.344	0.445
Distance + Distance:Elevation + Distance:Number of medium trees + Canopy cover:Number of medium trees + Number of large trees:Number of medium trees + Number of large trees:Proportion branching type B + Proportion branching type B:Proportion branching type C&D + Transect	0.05	0.07	0.320	0.410
Distance + Distance:Elevation + Distance:Number of medium trees + Canopy cover:Number of medium trees + Number of large trees:Number of medium trees + Transect	0.19	0.07	0.274	0.375
Distance + Distance:Elevation + Distance:Number of medium trees + Canopy cover:Number of medium trees + Number of large trees:Number of medium trees + Proportion branching type B:Proportion branching type C&D + Transect	0.58	0.05	0.296	0.405
Distance + Distance:Elevation + Distance:Number of medium trees + Number of large trees:Number of medium trees + Transect	0.61	0.05	0.216	0.322

Models are ranked using AICc. Model fit is represented by marginal *R*^2^ ($R_{{\text{m}}}^{2}$) and conditional *R*^2^ ($R_{{\text{c}}}^{2}$). Variable abbreviations are listed in Table 1

**Table 4:** Results of the fixed-effect parameters in the final GLMM explaining variation in hornbill presence-absence

Parameter	Estimate	SE	*z*	*P*
Intercept	− 2.242	0.534	4.164	< 0.001
Distance	0.953	0.398	2.378	0.017
Number of medium trees: distance	− 1.070	0.464	2.287	0.022
Distance: elevation	0.679	0.359	1.876	0.061
Number of large trees: number of medium trees	0.647	0.351	1.829	0.068
Proportion branching type B: proportion branching type C&D	0.808	0.510	1.571	0.116
Number of large trees: proportion branching type B	− 0.792	0.520	1.511	0.131

## Discussion

The estimated population size of 2673 individuals ± 21.3% CV within the whole Northwest Panay Peninsula is greater than the 2001 IUCN global population estimate of 1800 individuals. There may be several reasons for this difference. The IUCN estimate was derived from various analyses of surveys and records by BirdLife International in 2001, and is now considerably out-dated (BirdLife International 2020). Secondly, our survey is the first time that a survey has been undertaken across the whole Northwest Panay Peninsula, rather than at focal areas such as Sibaliw research station at the Park’s centre, and therefore our estimate, which encompasses data from previously unrecorded areas, may be more representative of the species across its range. The population size estimated here is complemented by previous estimates from the Central Panay Mountain Range (750–1500 breeding pairs; Klop et al. 2000) and by the Philippine Biodiversity Conservation Foundation’s recent estimate of 3564 hornbills across three forest blocks in Negros (Chavez 2020): 1580 individuals in North Negros Natural Park (708.3 km^2^); 532 in Mount Kanla-on Natural Park (245.6 km^2^); and 1452 in Balinsasayao Twin Lakes Natural Park (80.2 km^2^; 290–1762 m elevation; Chavez 2020). The overall lowland forest density estimate of 14 individuals/km^2^ (cited in BirdLife International 2020) from the Negros study is commensurate with that estimated in primary forest of the Northwest Panay Peninsula (17.8 individuals/km^2^). However, our population estimate for the NWPPNP exceeds these single landscape population estimates, suggesting that the area could be a particular stronghold of this species.

The density estimates for different habitat strata within this study suggest that forest cover with minimal disturbance is especially important to the Visayan Hornbill, with significantly higher density estimated in primary forest and it not being recorded in non-forest habitats in this study. This is supported by the different estimated population sizes of this species within the Natural Parks on Negros (Chavez 2020). While the largest population size was estimated in the geographically largest area, North Negros Natural Park, encounter rates there were lower than in Mt Kanla-on. Within North Negros Natural Park, only ~ 20% is forested, with some old-growth (BirdLife International 2021b). In comparison, Mt Kanla-on Natural Park has a smaller area, but about half is forested (BirdLife International 2021c). It is unclear from the Negros study how many sightings were recorded within primary or secondary forest.

As the GLMM demonstrates, hornbill presence in the NWPPNP is associated positively with distance from the Park’s boundary, as a proxy for human access and activities. This suggests that the species may prefer interior forest areas with less disturbance. However, increasing distance from the Park’s boundary becomes less important in explaining hornbill presence with increasing number of medium-sized trees, suggesting that hornbills are more likely to be found in areas of better and recovering forest nearer the Park’s boundary than would otherwise be expected based on distance from sources of anthropic activity alone. It is encouraging that areas of better forest near human settlements may help offset impacts associated with proximity to the Park, while also including trees of potentially suitable nesting size (Klop et al. 2000).

While not significant statistically, the positive effects of large trees and distance on the probability of hornbill presence increased with increasing numbers of medium-sized trees and at higher elevations, respectively. Larger trees indicative of primary forest tended to be found further from the Park’s boundary and at higher elevations. This reflects the fact that in general across this landscape, the least accessible forest (high-elevation and further from the boundary) is the least disturbed. This is possibly due to the increased difficulty of accessing such areas for logging. As found in our study, the lower population density within North Negros Natural Park was thought to be because the Park has fewer tall, mature trees suitable for hornbills (Chavez 2020). This may also explain why there is a relatively large population in the NWPPNP, where a high density of large and mature trees remains, based on our habitat survey.

Other hornbill species have shown similar responses to reduced forest quality. In protected area forests of Arunachal Pradesh, India, five hornbill species and the fruiting trees they use were found at reduced abundance in heavily disturbed forests (Naniwadekar et al. 2015). In urban-forest mosaics of KwaZulu-Natal, occupancy modelling showed that large trees had a positive influence on Trumpeter Hornbill (*Bycanistes bucinator*) presence, while human presence negatively influenced its detection probability (Chibesa and Downs 2017). Holbech et al. (2018) identified two small-bodied hornbill species in Ghana that are not subject to hunting pressure that have seen population declines, suggesting limited resilience to forest degradation (Holbech et al. 2018). In Ghana, while the versatile West African Pied Hornbill (*Lophoceros semifasciatus*) persists in fragmented forests, this fragmentation was thought to be a factor driving significant population declines (Holbech et al. 2018).

Even when hornbills can feed successfully in disturbed forest, they require large tree cavities for nesting (Klop et al. 2000; Marsden and Pilgrim 2003; Española et al. 2016). Consequently, they often breed at higher densities in primary forests (Española et al. 2016) and, as long-lived species, can show considerable time-lags in population decline following forest disturbance (Marsden and Pilgrim 2003). This reliance on large trees in primary forests is supported by our habitat data, in which hornbill presence is associated with the positive interaction between medium-sized and large trees.

Despite protection of the NWPPNP and its large primary forest area, illegal logging still occurs (PhilinCon 2020), and the Park lost 2% forest cover between 2000 and 2018 (Abrahams et al., unpublished data). However, forest loss has been worse in unprotected areas over the Visayan Hornbill extant range, with 4.6% of unprotected forest cover across Panay and Negros lost within the same period (Abrahams et al., unpublished data). Alongside illegal threats, the Park is currently threatened by a 0.25 km^2^ hydropower development within its borders at Malay, Aklan (Antique Union for Conservation 2020). Therefore, future conservation efforts need to target and protect this Natural Park, a key stronghold for this species, and specifically the primary forest within this Park.

A further factor affecting Visayan Hornbill populations is hunting (BirdLife International 2020). Several studies have suggested that hunting may have overtaken deforestation as the greatest threat for bird species across Southeast Asia (e.g. Sreekar et al. 2015; Harrison et al. 2016). Although the NWPPNP is a protected area, the Visayan Hornbill is still hunted inside the Park (PhilinCon 2020). In fact, signs of hornbill hunting (e.g. plucked feathers), rare before the coronavirus pandemic, have been witnessed since its start in 2020 (R.A. Santillan pers. obs.). For some species, hunting for the international wildlife trade has a critical impact, but many more are pressured by domestic consumption (Sreekar et al. 2015). In 1998, Klop et al. (2000) recorded hunting of the Visayan Hornbill in the Northwest Panay Peninsula for subsistence and capture for the pet trade. Also, legs, feathers and bills of Visayan Hornbills are converted into tourist souvenirs sold on nearby Boracay Island (C.J. Schwarz pers. obs.). Hunting pressure may explain cases in which hornbills do not utilise certain forests or fragmented habitats (Holbech et al. 2018) and plantations, despite such disturbed areas containing suitable food resources (Marsden and Pilgrim 2003). This idea is supported by conversations with local people around the NWPPNP, who suggest that poaching pressure is the reason that hornbills, which used to visit the villages in the daytime, no longer do so (C.J. Schwarz pers. obs.). Supporting this further, increasing distance from the Park boundary (and, therefore, distance from human settlements and sources of hunting pressure) was also the only fixed main effect that explained variation in hornbill presence. Therefore, hunting pressure may explain why hornbills were only observed in primary and secondary forest in this study. A recent study in Mindanao, Philippines, suggests that while wildlife was traditionally hunted for sustenance, the more recent drivers of hunting within protected areas are poverty and lack of long-term livelihood options (Tanalgo 2017). Further research is recommended on the drivers for Visayan Hornbill hunting within the NWPPNP.

Regarding modelling the hornbill distance data, hornbills were not detected < 20 m from points in primary forest, indicating undetected movement near points in this habitat, and reflected in habitat-specific detection functions best fitting the survey data. Movements in response to observers are more likely to remain undetected in areas of forest with taller, denser structures and canopies, and this possible violation of distance sampling assumptions (Buckland et al. 2001) has been reported from point count surveys of Mindoro Hornbill (*Penelopides mindorensis*; Lee 2005) and anecdotal experience with the focal species (A.D. Fernandez pers. comm.). While left truncation of these data can perhaps help mitigate this, our models and estimates became less reliable as a result. Whilst a settling down period has previously been recommended to reduce the impact of observer presence, a study in the Philippines has demonstrated that using a 10-min settling down period resulted in up to 3.48 times fewer detections of canopy frugivores than without (Lee and Marsden 2008). Therefore, we encourage field observers of *Penelopides* species to be especially mindful of undetected movements in denser forest habitats and its consequences to detection modelling.

## Conclusions

This study contributes empirically to the conservation assessment of this threatened species, providing strong evidence for re-assessing the global population size, and alongside the previous population estimates from Panay and Negros (see Klop et al. 2000; Chavez 2020). It also provides strong evidence that the Northwest Panay Peninsula, especially the protection of its Natural Park, supports a large population concentrated outside of the other significant tract of forest on Panay, the Central Panay Mountain Range (Klop et al. 2000; BirdLife International 2020), where it is considered, subjectively, fairly common (Alabado et al. 2009). As well, this study presents evidence of the species’ habitat associations, which should guide future landscape management within and beyond the protected area. Finally, these population data provide a robust baseline against which long-term monitoring can be measured, aligning with the proposed conservation action of replicating a hornbill monitoring scheme across Panay (BirdLife International 2020).

## Supplementary Information

**Additional file 1: Table S1.** Model fit measures for overall and habitat detection functions at different right truncation distances.

## Data Availability

The datasets supporting the conclusions of this article are available in the Open Science Framework repository, https://doi.org/10.17605/OSF.IO/J9AET, at https://osf.io/j9aet/.
