# Supplementary material for: Population assessment and habitat associations of the Visayan Hornbill (Penelopides panini) in Northwest Panay, Philippines
Source: Avian Res. 2021 Nov 29;12(1):67. doi: 10.1186/s40657-021-00303-3 (PMC8628822; doi:10.1186/s40657-021-00303-3)
Supplement: Supplementary file 1 — Additional file 1: Table S1. Model fit measures for overall and habitat detection functions at different right truncation distances. [file 40657_2021_303_MOESM1_ESM.docx]

**Additional file 1**

**Table S1. Model fit measures for overall and habitat detection functions at different right truncation distances..**

| **Truncation distance (m; ΔAIC) ^a^** | **Detection function** | **Goodness-of-fit probabilities, *P*^b^** | | | **EDR^c^ ± %CV** | ***g*(*y*)^d^ ± %CV** | **N ± %CV** |
| --- | --- | --- | --- | --- | --- | --- | --- |
|  |  | **K-S** | **CvM** | **Chi-square** |  |  |  |
| 105 (+9.49) | Overall | 0.245 | 0.500 - 0.600 | 0.040 | 68.5 ± 7.3 | 0.426 ± 14.6 | 2,564 ± 26.3 |
|  | PF | 0.287 | 0.600 - 0.700 | 0.901 | 67.9 ± 5.1 | 0.419 ± 10.2 | 1,758 ± 26.7 |
|  | SF | 0.476 | 0.500 - 0.600 | 0.176 | 105.0 ± 0.0 | 1.0 ± 0.0 | 444 ± 44.4 |
| 100 (+7.99) | Overall | 0.202 | 0.500 - 0.600 | 0.374 | 69.2 ± 8.4 | 0.479 ± 16.8 | 2,516 ± 27.6 |
|  | PF | 0.291 | 0.600 - 0.700 | 0.790 | 67.9 ± 5.1 | 0.461 ± 10.3 | 1,761 ± 26.7 |
|  | SF | 0.270 | 0.500 - 0.600 | 0.176 | 100.0 ± 0.0 | 1.0 ± 0.0 | 490 ± 44.4 |
| 95 (+0.56) | Overall | 0.202 | 0.400 - 0.500 | 0.171 | 65.3 ± 10.4 | 0.473 ± 20.9 | 2,586 ± 29.0 |
|  | PF | 0.260 | 0.500 - 0.600 | 0.251 | 68.3 ± 5.4 | 0.517 ± 10.7 | 1,737 ± 26.9 |
|  | SF | 0.210 | 0.300 - 0.400 | 0.268 | 95.0 ± 0.0 | 1.0 ± 0.0 | 371 ± 33.2 |
| 90 (+0.79) | Overall | 0.205 | 0.400 - 0.500 | 0.719 | 64.7 ± 9.9 | 0.517 ± 19.8 | 2,737 ± 28.1 |
|  | PF | 0.617 | 0.600 - 0.700 | 0.542 | 60.7 ± 10.2 | 0.455 ± 20.3 | 2,201 ± 32.0 |
|  | SF | 0.326 | 0.400 - 0.500 | 0.190 | 90.0 ± 0.0 | 1.0 ± 0.0 | 414 ± 33.2 |
| 85 (-0.08) | Overall | 0.132 | 0.400 - 0.500 | 0.292 | 65.1 ± 7.0 | 0.586 ± 13.9 | 2,434 ± 24.3 |
|  | PF | 0.473 | 0.600 - 0.700 | 0.706 | 62.9 ± 7.3 | 0.548 ± 14.6 | 1,954 ± 28.5 |
|  | SF | 0.163 | 0.200 - 0.300 | 0.091 | 85.0 ± 0.0 | 1.0 ± 0.0 | 357 ± 30.5 |
| 80 (+0.04) | Overall | 0.149 | 0.400 - 0.500 | 0.238 | 61.7 ± 12.6 | 0.595 ± 25.1 | 2,709 ± 32.0 |
|  | PF | 0.463 | 0.500 - 0.600 | 0.769 | 59.5 ± 13.5 | 0.554 ± 27.0 | 2,182 ± 36.5 |
|  | SF | 0.294 | 0.300 - 0.400 | 0.091 | 80.0 ± 0.0 | 1.0 ± 0.0 | 403 ± 30.5 |
| 60 (-0.93) | Overall | 0.081 | 0.150 - 0.200 | - | 54.3 ± 7.2 | 0.820 ± 14.5 | 2,722 ± 28.4 |
|  | PF | 0.122 | 0.400 - 0.500 | 0.490 | 60.0 ± 0.0 | 1.0 ± 0.0 | 1,729 ± 29.7 |
|  | SF | 0.193 | 0.200 - 0.300 | 0.102 | 60.0 ± 0.0 | 1.0 ± 0.0 | 501 ± 36.7 |

^a^ ΔAIC of overall and habitat models; +ve (AIC_overall_ > AIC_habitat_), -ve (AIC_habitat_ > AIC_overall_).

^b^ K-S = Kolmogorov-Smirnov test, CvM = Cramér-von Mises (with cosine weighting function).

^c^ EDR = Effective detection radius.

^d^ *g*(*y*) = Probability of detection (within distance *y*). PF = Primary Forest. SF = Secondary forest
